# Inside the Tumor: Decoding the Feline Mammary Tumor Microenvironment and Its Prognostic Value—A Review

**DOI:** 10.3390/vetsci12100959

**Published:** 2025-10-08

**Authors:** Joana Rodrigues-Jesus, Ana Canadas-Sousa, Hugo Vilhena, Patrícia Dias-Pereira

**Affiliations:** 1 Department of Pathology and Molecular Immunology, School of Medicine and Biomedical Sciences, ICBAS-UP, University of Porto, 4050-313 Porto, Portugal; jjoanarodrigues@gmail.com (J.R.-J.); canadas.ana@gmail.com (A.C.-S.); 2 Associated Laboratory for Green Chemistry (LAQV), REQUIMTE, University of Porto, 4050-313 Porto, Portugal; 3 Vasco da Gama Research Centre (CIVG), Department of Veterinary Sciences, Vasco da Gama University School (EUVG), 3020-210 Coimbra, Portugal; hcrvilhena@hotmail.com; 4 Department of Veterinary Clinics, School of Medicine and Biomedical Sciences, ICBAS-UP, University of Porto, 4050-313 Porto, Portugal; 5 Animal and Veterinary Research Centre (CECAV), University of Trás-os-Montes e Alto Douro, 5000-801 Vila Real, Portugal; 6 Associate Laboratory of Animal and Veterinary Sciences, AL4AnimaLS, 1300-477 Lisbon, Portugal

**Keywords:** tumor microenvironment, necrosis, fibrosis, angiogenesis, adipose tissue, tumor-infiltrating lymphocytes, tumor-associated macrophages, extracellular vesicles, epithelial-to-mesenchymal transition, feline mammary tumors

## Abstract

**Simple Summary:**

The tumor microenvironment has become a central focus in cancer research, offering new insights into cancer behavior and showing strong potential for improving cancer treatment. However, in veterinary oncology, specifically feline mammary tumors, research on the topic lags far behind. This review summarizes current knowledge on key elements of the tumor microenvironment in the feline mammary tumors, including tumor necrosis, fibrosis, angiogenesis, adipose tissue, tumor-associated inflammation, extracellular vesicles, and epithelial–mesenchymal transition. Although studies in cats remain limited, insights from human breast cancer are frequently drawn, creating a combined perspective that may help drive new research into the diagnostic, prognostic, and therapeutic potential of the tumor microenvironment in feline mammary tumors.

**Abstract:**

The tumor microenvironment (TME) comprises neoplastic and stromal cells, and extracellular matrix elements, all engaging in a complex interplay that ultimately dictates tumorigenesis, cancer progression, and therapeutic response. While extensive research on the TME has been conducted in human oncology, data on its veterinary counterpart, particularly in feline mammary tumors (FMTs), are still scarce. In this review, we explore current understanding of feline mammary carcinoma (FMC) microenvironment, focusing on tumor necrosis, fibrosis, angiogenesis, adipose tissue tumor-associated inflammation, extracellular vesicles, and epithelial–mesenchymal transition (EMT) and their prognostic implications. In FMC, remodeling of collagen fibers, cancer-associated fibroblasts (CAFs), regulatory T cells (Tregs) and elevated serum leptin have been associated with poor prognosis, whereas stromal cytotoxic T cells correlate with more favorable outcomes. By contrast, findings on necrosis and pro-angiogenic factors remain inconsistent, and research on extracellular vesicles (EVs) is still in its early stages. This review presents insights from human breast cancer (HBC) that further support and elucidate the potential relevance of these TME components. As FMCs are highly aggressive tumors, a deeper understanding of their microenvironment could not only improve prognostic accuracy but also uncover novel therapeutic targets. Furthermore, due to their similarities, FMCs offer a potential valuable spontaneous model for HBC, particularly for the aggressive triple-negative phenotypes.

## 1. Introduction

The concept of tumor microenvironment (TME) originated in the late 19th century when Stephen Paget, while studying the distribution of metastatic breast cancer, alluded that neoplastic malignant cells behave like seeds, dispersing throughout the body, but only thriving in a suitable environment [1]. Regardless, it was not until the late 20th century that researchers began to focus on the tumor milieu [2]. The TME is composed not only of neoplastic cells, but also of stromal components, such as immune cells, fibroblasts, vascular cells, adipocytes and extracellular matrix (ECM) elements. All these components engage in a complex interaction with tumor cells, taking on a supportive or inhibitory role in cancer progression [2,3,4,5].

The great plasticity and adaptability of neoplastic cells often allow them to evade the host surveillance mechanisms and to selected therapeutic regimes. Indeed, resistance to cancer therapy remains a major challenge in cancer treatment, and the TME takes on an active role modulating the therapeutic response and constantly adapting to its milieu [6,7]. Understanding the TME constituents and the multifaceted interactions between tumor cells and the surrounding microenvironment might enable the identification of new and more effective therapeutic targets and, consequently, the development of combined therapies that are able to overcome the resistance of current treatments.

Although still limited, research on feline mammary tumor (FMT) microenvironment has been steadily growing in recent years. In this review, we explore some of the key components of the TME and their prognostic implications—specifically tumor necrosis, fibrosis and angiogenesis, adipose tissue, tumor-associated lymphocytes (TIL) and macrophages (TAM)—as well as less explored elements such as other immune cell populations, immune phenotypes and checkpoints, and extracellular vesicles. We also address the epithelial–mesenchymal transition as a key process to tumor invasion. Mammary carcinomas in felines and human breast cancer (HBC) present similar clinical behavior, metastatic pattern, histopathological features and molecular subtypes. Building on this, feline mammary carcinoma (FMC) has been proposed as a good spontaneous model for studying tumor progression, TME and therapeutic strategies in HBC—particularly in the more aggressive triple-negative mammary carcinomas, which, while rare in humans, are common in cats [8,9,10,11,12,13,14,15,16]. Thus, relevant findings from HBC literature are provided to support our discussion. This review seeks to underscore the importance of the TME and to encourage further investigation into its diagnostic and therapeutic potential in FMT.

## 2. Tumor Necrosis

**Figure 1:**
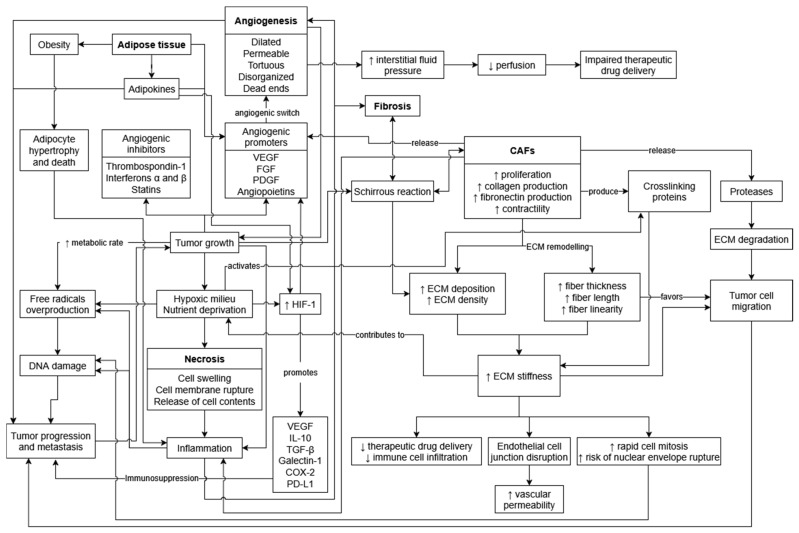
Diagram summarizing the complex interplay between different elements of the tumor microenvironment.

Cell death can occur as part of physiological or pathological mechanisms, the latter taking place in response to irreversible damage to the cell. Necrosis, also known as uncontrolled cell death, often involves groups of cells and happens following external irreversible cell damage, resulting in cell swelling with rupture of the cell membrane, release of cellular contents and consequent inflammatory reaction [17,18]. This type of cell death is often encountered in malignant solid tumors and it appears to be associated with aggressive pathological features and poor prognosis [19]. As tumors grow, the vascular supply often fails to keep pace with tumor growth, ensuing hypoxia and nutrient deprivation in the central tumor region, ultimately resulting in necrosis (Figure 1). Additionally, the hypoxic microenvironment, combined with the increased metabolic rate of neoplastic cells and the inflammatory response triggered by necrosis, promotes overproduction of free radicals, including reactive oxygen species (ROS). This oxidative stress drives additional DNA damage and mutagenesis, thereby contributing to tumor progression and metastasis [20,21,22]. Notwithstanding, the mechanisms underlying tumor necrosis are still not completely understood, as it is unclear whether it results from tumor progression or directly contributes to tumor aggressiveness [23]. Lately, several different mechanisms of programmed necrosis have been recognized; however, their role during tumor development remains incompletely deciphered [23,24].

In veterinary medicine, the percentage of tumor necrosis is used in several tumor grading systems, including canine soft tissue sarcomas, canine splenic hemangiosarcoma, canine pulmonary carcinoma, and feline and canine osteosarcoma [25]. It is reasonable to assume that only histological necrosis is considered; however, the overall lack of detail in the described methods makes it unclear whether the assessment was performed during macroscopic and/or microscopic evaluation. Additionally, variations in trimming techniques, coupled with the frequent exclusion of macroscopic necrotic areas, may introduce bias into the microscopic evaluation, given that the resulting histological section may not accurately represent the entirety of the tumor [25,26]. Although necrosis is not a parameter included in the histological grading criteria of mammary tumors, it is a frequent histological finding in both humans and felines alike, being present in 42.2–68.4% and 55.6–89.8% of the malignant mammary cases reported in the literature, respectively [15,27,28,29,30,31,32,33,34,35]. Additionally, in the current official classification system for mammary tumors in domestic animals, it is recommended that the haphazardly distributed necrotic foci should be considered a criterion for malignancy, contrary to ischemic central necrosis, which can be observed in large benign lesions [36]. Although most available literature on FMC lacks specific details regarding necrosis assessment, the evaluation likely included overall necrosis, without distinction of type or pattern [27,28,34,37,38,39]. Recently, our group addressed this matter in a study that described different histological necrosis patterns and offered detailed semi-quantitative and quantitative methods for the evaluation of overall necrosis in FMT [35].

In invasive HBC, the presence of necrosis has been associated with larger tumors, higher histological grade and more prominent inflammatory infiltrate [33,40,41,42]. Furthermore, cases with extensive necrosis had a higher probability of treatment failure than those with slight or moderate necrosis in one study. Interestingly, the same study observed a significant relationship between comedo necrosis and the presence of multicentric neoplastic foci distant from the primary tumor [30]. Nevertheless, regarding the independent prognostic value of tumor necrosis, results are not consensual among authors. While Gilchrist and colleagues (1993) revealed an independent association between tumor necrosis and disease-free interval (DFI) and survival, Carlomagno and co-workers (1995) failed to find a significant relation between necrosis and survival after adjusting for other clinicopathological features [41,43]. In a similar manner, Carter and co-workers (1978) observed a significant correlation between necrosis and the presence of lymph node metastasis, whereas Fisher and colleagues (1978) did not observe an association between the same variables [30,42].

Similarly, the influence of tumor necrosis has yielded conflicting results regarding FMC prognosis (Table 1). In one earlier study, tumor necrosis was identified as an independent survival prognostic factor reported in categories ranging from no necrosis to some necrosis, ≈30%, 60% and ≥90% [39]. More recently, distinct studies evaluated the presence and the extent of histological necrosis and failed to find a significant relationship with clinical outcomes of cats with malignant mammary tumors [28,35,37]. On the other hand, a different study using a 25% cutoff found a significant association with prognosis [27]. Furthermore, in another study, queens with extensive necrosis presented a median tumor-specific survival (TSS) of 8 months and a median DFI of 7 months, compared to a median of 13 and 12 months, respectively, in cats with necrosis of isolated cells [38]. Likewise, queens with ischemic central necrosis displayed shorter overall survival (OS) and DFI (6 and 5 months, respectively), compared to those without this pattern of necrosis (13 and 10 months, respectively) [35]. However, survival analyses did not reach statistical significance [35,38].

In a related context, the presence of ulceration, infiltrative behavior and a more pronounced chronic inflammation has been positively associated with necrosis [39]. Also, a recent approach revealed that total (intratumoral and stromal) cytotoxic CD8+ tumor-infiltrating lymphocytes (TILs) were significantly associated with the presence of necrosis in FMC [34]. As previously alluded, the conflicting results obtained regarding the prognostic value of tumor necrosis in FMC could be related to different definitions and variations of the methodological assessment of this morphological feature.

Although ischemic central necrosis can occur in benign lesions, studies on human solid neoplasms have established an association between hypoxia and poor prognosis [23]. The hypoxic tumor environment induces overexpression of hypoxia-inducible factor-1 (HIF-1), which subsequently promotes the upregulation of immunosuppressors such as vascular endothelial growth factor (VEGF), IL-10, transforming growth factor beta (TGF-β), galectin-1, COX-2, and programmed death ligand 1 (PD-L1), thereby contributing to tumor progression and metastasis [48,49]. However, while overexpression of HIF-1α has been reported in FMC by Chen and colleagues (2020), no significant associations were found between HIF-1α and clinicopathological features, and there was no correlation with VEGF overexpression. Moreover, despite obtaining a statistically significant difference in the Kaplan–Meier survival curves of cats with and without HIF-α overexpression [27], the FMC group without HIF-1α overexpression had a reduced sample size, which may have affected the reliability of these findings.

## 3. Tumor Angiogenesis

Angiogenesis is essential for tumor growth and progression. In their initial stages of development, solid tumors are typically avascular and may remain dormant for long periods of time [50,51]. During this time, there is a balance between angiogenic promoters (i.e., VEGF, fibroblast growth factors (FGF), platelet-derived growth factors (PDGF) and angiopoietins) and inhibitors (i.e., thrombospondin-1, interferons α and β, and statins such as angiostatin, endostatin, canstatin and tumstatin) [52,53]. However, in order for the tumor mass to expand beyond its critical size, a proper blood supply is required to deliver oxygen and nutrients to the neoplastic cells, which otherwise undergo starvation, ensuing apoptosis and/or necrosis (Figure 1) [50,52]. The angiogenic switch occurs when the net balance between pro- and anti-angiogenic factors shifts in favor of pro-angiogenics, eliciting the development of blood vessels in the tumor [51,52]. These tumor-associated vessels are often more dilated, permeable, tortuous, and disorganized than normal vasculature [50,54,55], and sometimes even form dead ends [53]. Furthermore, although the continuously expanding tumor-associated vascular network and its increased permeability [53,54] facilitate dissemination of neoplastic cells and consequent metastasis [51,52,56], it also impairs therapeutic drug delivery due to a diminished perfusion resulting from elevated interstitial fluid pressure and irregular vascular irrigation [56].

In FMCs, two studies reported VEGF overexpression in 75.0–77.8% of the cases [27,45], with positive immunostaining being observed in tumor cells and, at times, in stromal cells [27,44]. Accordingly, VEGF positivity was observed in the tumor epithelium of 100%, the endothelium of 79.1% and the stroma of 58.3% of a total of 48 FMCs [57]. Comparable findings were reported in a cohort of 50 cats, in which a weak to strong positive VEGF-A immunoreactivity was present in the tumor cells and TILs of 95% and 51% of the mammary carcinomas, respectively [58]. VEGFR-2 expression was recorded in the tumor cells of 100%, the endothelial cells of 37.5% and the stromal cells of 18.7% of cases [57]. Likewise, VEGFR-1 and VEGFR-2 positivity was also found in cancer cells of 19% of the tumors (for both receptors), and in TILs of 22% and 24% of cases, respectively [58]. VEGF overexpression was associated with several clinicopathological features, including histological classification, stromal response, squamous differentiation and lymphovascular invasion (LVI) [27]. Furthermore, a higher percentage of VEGF immunoreactivity was present in papillary and solid mammary carcinomas, and an increased proportion of positive cells was also detected in FMCs Nottingham grade III compared to grades I and II [44]. These findings were further supported by a later study identifying an increased VEGF immunoreactivity in FMCs Nottingham grade III compared to grade I [59]. On the other hand, unlike Chen and colleagues (2020), Millanta and co-workers (2002) found no association between VEGF expression and LVI [27,44]. Interestingly, compared to a negative or weak expression, a strong VEGF-A immunoreactivity in TILs was associated with increased serum VEGF-A levels. Additionally, a strong positivity of VEGFR-2 in TILs was also correlated with higher levels of serum VEGF-A and VEGFR-1, compared to their negative or weak counterparts [58]. Upon univariate survival analysis, an early study on FMCs reported a significant relationship between VEGF expression (median 72.1% cutoff) and survival, with median survival times of 18.4 and 14.2 months in queens presenting a VEGF positivity percentage below and above the cutoff value, respectively [44]. In contrast, later studies found no direct association between VEGF expression and survival [27,45], as summarized in Table 1. These findings suggest that VEGF may play an important role in FMC, with TILs also possibly influencing the systemic levels of these angiogenic factors, thereby promoting angiogenesis and subsequent tumor progression.

Curiously, Millanta and colleagues (2002) found no correlation between VEGF expression in FMCs and tumor microvessel density (MVD) [44]. On the other hand, another study observed a progressively higher MVD in FMCs with increased VEGF expression, revealing a correlation between both features [59]. Moreover, even though queens with mammary carcinomas presenting higher MVD exhibited poorer survival times, no statistically significant prognostic value was found [44]. Although findings regarding the correlation between VEGF expression and MVD are inconsistent and no statistically significant prognostic value has been demonstrated for the latter, available evidence suggests that high MVD may be linked to poor prognosis.

In a related study addressing the importance of lymphangiogenesis in FMT, there was an overall higher number of lymphatic vessels in extratumoral areas compared to intratumoral regions. No differences were found between the number of VEGFR-3-negative lymphatics in malignant tumors and non-malignant mammary tissues (normal mammary glands and benign tumors). Notwithstanding, the number of intratumoral VEGFR-3-positive lymphatic vessels significantly increased with malignant tumor invasiveness. Additionally, higher VEGF-C immunostaining values were observed in the intratumoral region of malignant tumors than in extratumoral areas. Despite this, no differences were found in VEGF-C expression when comparing normal mammary glands, benign tumors and malignant tumors, or when assessing tumor invasiveness. Thus, these data provide no evidence of active lymphangiogenesis in feline malignant tumors, indicating that the tumor cells likely spread through pre-existing lymphatic vessels, rather than newly formed ones [60].

## 4. Tumor Fibrosis

Fibrosis is defined as an excessive buildup of extracellular matrix (ECM) components, primarily collagen, resulting from fibroblast hyperproliferation that ensues tissue injury [61,62]. After the removal of necrotic material and inflammatory exudate by macrophages, granulation tissue forms, which is subsequently replaced by fibrous connective tissue and ultimately results in a scar [63]. Over the years, tumors have been referred to as “wounds that do not heal”, given that chronic fibrosis is a risk factor for cancer development [64,65], which, in turn, can trigger a scirrhous reaction [66,67], thus entering a vicious cycle. When compared to normal tissue, tumor-associated ECM is biochemically different, and tumor stroma is stiffer [61,68,69]. This is particularly true in cases of breast cancer, which can be ten times stiffer than normal breast tissue [61]. Furthermore, several studies have demonstrated that breast density is a risk factor for breast cancer [70].

Tumor stiffness is essential during tumorigenesis, as it counteracts the tensile strength exerted by the host’s normal tissue, enabling the neoplastic mass to expand and displace the surrounding tissues [71]. The tumor’s ECM undergoes constant remodeling, typically acquiring an increased rigidity mainly due to excessive matrix deposition, contraction and crosslinking (Figure 1) [68,72,73]. This stiffened ECM not only facilitates malignantly transformed cell proliferation, mobility and metastasis, but also hinders therapeutic drug delivery and immune cell infiltration [64,69], by altering chemotactic gradients [69] and physically impairing their migration [74]. It is also believed that a stiffer matrix heightens the likelihood of genome instability by promoting rapid cell mitosis and increasing the risk of nuclear envelope rupture and DNA damage [69]. Furthermore, greater matrix stiffness and abnormal ECM characteristics contribute to tumor hypoxia and disrupted endothelial cell junctions, resulting in increased vasculature permeability, which has been discussed in the previous section [69]. On a cellular level, cancer-associated fibroblasts (CAFs) are key components of the TME, constituting a substantial portion of the tumor volume and playing a central role in ECM production [69]. Compared to normal fibroblasts, CAFs present greater proliferation rates [64,74] and produce higher amounts of collagen and fibronectin, thus resulting in increased ECM deposition, density and stiffness [68,73]. Cancer-associated fibroblasts also exhibit higher contractility and can mechanically remodel the ECM by straightening, bundling and reorienting collagen fibers, thus creating a heterogeneous stroma that favors tumor cell migration [72]. Accordingly, one study in breast cancer observed that, as there was a progression from normal mammary tissue to ductal carcinoma in situ and to invasive ductal carcinoma, the associated collagen fibers became increasingly thicker, longer and more linear [75]. The hypoxic TME induces the activation of crosslinking proteins such as lysyl oxidase, which CAFs can also produce, reinforcing matrix stiffening [68,72]. On the other hand, degradation of the ECM does likewise occur during tumor development, given that CAFs, neoplastic cells and TAMs can release proteases such as matrix metalloproteinases [68,72]. In addition, CAFs can modulate the tumor-associated immune response through several mechanisms, such as the secretion of pro-inflammatory cytokines [64,74]. Several CAF markers, such as fibroblast activation protein (FAP), α smooth muscle actin (α-SMA), PDGF receptor, fibroblast-specific protein-1 (FSP-1) and podoplanin have been evaluated in HBC, with significant associations found in patient outcomes [76,77,78,79,80,81,82,83,84,85]. These findings support the predominantly tumor-promoting role of CAFs during breast cancer development and progression, as their presence was more frequently linked to poor prognosis [77,80,81,82,83,84,85].

In the same context, tumor-stroma ratio (TSR) has been under the scope of several studies in HBC, being recognized as a relevant morphological feature to include in the diagnostic routine. Research has determined that a TSR cutoff of 50% provides the greatest discriminative power for predicting prognosis in HBC [86]. Accordingly, patients with stroma-rich tumors (TSR > 50%) present significantly worse survival and DFI than those with stroma-poor tumors (TSR ≤ 50%), independently of other clinicopathological features [86,87,88,89]. Recently, to further improve homogeneity between studies and reproducibility, Hagenaars and colleagues (2022) reviewed existing literature and outlined a standardization of the TSR scoring method for HBC in both microscopic and digital analysis. For the microscopic assessment, the percentage of stroma (in 10% increments) is assessed on the HE slides, in the field of view (10× objective) with the largest stromal component, while containing neoplastic cells on all four sides of the image [90]. Notably, the TSR offers a straightforward, cost-effective and reproducible approach that is easy to apply in the diagnostic routine [86,87,88,90]. Despite the importance of TSR in human oncology, to the authors’ best knowledge, this method has yet to be applied in veterinary oncology, viz in FMT.

One study in canine mammary tumors (CMTs) observed larger areas of fibrosis in tubular carcinomas, while benign mixed tumors presented lower fibrosis. Furthermore, the study reported a statistically significant difference between benign and malignant neoplasms, suggesting an association—similar to that observed in HBC—between increased stroma and worse prognosis [91]. Notwithstanding, the methodology lacks detail, which may limit reproducibility by independent researchers. Another study found that dogs presenting mammary carcinomas with thick, long, straight collagen fibers and a poorly defined tumor-stroma boundary had poorer survival outcomes [92]. The same group later assessed the prognostic potential of collagen features in FMCs and found similar results [46]. Accordingly, shorter survival and DFI times were observed in cats with tumors displaying higher mean collagen density and long, thick and straight collagen fibers. Additionally, FMCs with poorly defined tumor-stroma boundaries were associated with worse survival outcomes than those with well-defined boundaries, as summarized in Table 1. Through multivariate analysis, the authors developed and proposed a model for the best OS predictive value, including Mills’ histological grade, surgical margins, tumor-stroma boundary score, collagen fiber length and collagen fiber straightness. As for DFI, the proposed multivariate model incorporated Mills’ histological grade, surgical margins, collagen fiber width and tumor-stroma boundary score [46]. Another study reported a significant association between the 1-year TSS and stromal response (i.e., none to mild, peritumoral or intratumoral) [27]. Regarding the assessment of CAFs in FMC, in a cohort of 50 cats, a high α-SMA-positive CAF score was observed in 68% of cases. Furthermore, a significant relationship was described between the presence of such high immunoreactivity and several aggressive features, such as increased mitotic count, positive nodal status, presence of LVI, distant metastasis and tumor recurrence. Upon survival analysis, cats with high α-SMA-positive CAF expression had a significantly shorter TSS and DFI, presenting a mean TSS and DFI of 348 and 305 days, respectively, compared to a mean TSS and DFI of 558 and 611, respectively, in the low immunoreactivity group [47]. Even though these studies included small sample sizes, their findings support the idea that both ECM and CAFs may play an important role in modulating tumor behavior, similar to that observed in humans, influencing subsequent clinical outcomes. Therefore, these are potentially important morphological features to evaluate during routine diagnosis and could also serve as potential therapeutic targets.

## 5. Adipose Tissue

As close human companions, domestic animals are often exposed to similar extrinsic risk factors for cancer, making them valuable models for studying the disease. Obesity, a well-recognized risk factor for several types of cancer, including breast cancer, is also highly prevalent in companion animals. In cats, up to 63% of the population is estimated to be overweight and obese [93,94,95]. As a consequence of obesity, adipocytes become enlarged and undergo cell death, triggering an inflammatory reaction that underlies the classification of obesity as a chronic systemic inflammatory condition (Figure 1) [96]. Moreover, adipose tissue in the breast tissue is highly vascularized and secretes angiogenic promoters, which, together with the pro-inflammatory microenvironment of obesity, contribute to angiogenic dysregulation within the TME [97]. During breast carcinogenesis, tumor cells trigger lipolysis and drive lipocytes to acquire a cancer-associated phenotype (CAA). CAAs and cancer cells engage in a bidirectional crosstalk, reprogramming glucose, lipid, and amino acid metabolism pathways in ways that promote tumor progression and metastasis [98].

Leptin is an adipokine best known for appetite suppression [95]. Clinical HBC studies suggest it also interacts with tumor cells, and may also be involved in the regulation of the immune system [96] and tumor angiogenesis [97]. One study on FMCs evaluated leptin and its receptor (ObR) in both tumor tissues and corresponding serum samples. In tumor tissues, leptin and ObR were expressed in the stromal cells of 72.2% and 18.2% of tumors, respectively, and a mean 81% and 87.6% of inflammatory mononuclear cells were also positive for leptin and ObR, respectively. Leptin overexpression was observed in luminal B and triple-negative carcinomas, while ObR expression was increased in luminal B tumors. Cats with FMCs had a significantly lower free leptin index (leptin/ObR ratio) than healthy controls, due to increased ObR and possibly reduced soluble leptin levels. Serum leptin was decreased in luminal B and HER2-positive carcinomas, but increased in luminal A tumors. Additionally, higher leptin levels were associated with tumor ulceration and shorter DFI (Table 1), while elevated ObR levels were linked to smaller tumors and ER-negative status. Serum ObR was also positively correlated with serum cytotoxic T lymphocyte-associated antigen 4 (CTLA-4), tumor necrosis factor α (TNF-α), programmed death protein-1 (PD-1), and programmed death ligand-1 (PD-L1), while showing an inverse correlation with ObR expression in tumor tissues [32]. These results indicate that leptin and its receptor may actively contribute to FMC pathogenesis by promoting immunosuppression and tumor aggressiveness. They also highlight leptin as a potential therapeutic target in luminal B and triple-negative subtypes, and support the use of serum leptin and ObR as possible diagnostic biomarkers of FMCs.

## 6. Tumor-Associated Inflammation

The original cancer immunosurveillance hypothesis was first proposed in the late 1950s and postulated that the immune system could detect and eliminate transformed cells [99,100]. However, this theory was abandoned in subsequent years due to insufficient supporting evidence. In 2002, Dunn and colleagues introduced the currently accepted tumor immunoediting hypothesis, consisting of three stages: elimination, equilibrium and escape [101,102].

While inflammation, specifically chronic inflammation, might be triggered by the growing mass of transformed cells disrupting the normal tissue homeostasis, it can also precede carcinogenesis [3], and has been established as a risk factor in the development of breast cancer in women [103]. Accordingly, although immune cells are a consistent finding in normal breast tissues [104], increased density of inflammatory cells has been observed in premalignant mammary lesions [105], suggesting a role in cancer initiation.

A summary of existing literature on FMC encompassing an evaluation of the tumor-associated inflammation is shown in Table 2. Early research on FMC identified chronic inflammatory infiltrate (i.e., lymphocytes and plasma cells) at the periphery of most tumors, but found no correlation between the amount of immune infiltration and the survival times [106]. On univariate analysis, the same authors later observed an association between increased chronic inflammation and poorer survival; however, significance was lost on multivariate analysis [39]. Nonetheless, Weijer and Hart [39] did not elaborate on the methodology used for this evaluation, describing it merely as the quantity of chronic and acute inflammatory infiltrate, which makes it difficult to replicate and critically assess their work. Ensuing studies have mostly overlooked this morphological feature of FMT until more recently. Wiese and co-workers (2012) evaluated the presence of tumor-associated inflammation as follows: negligible or absent (score 0), few lymphoid aggregates (score 1+), moderate lymphoid aggregates (score 2+), and brisk lymphoid aggregates or infiltrates associated with more than 50% of the tumor (score 3+). While few to moderate lymphoid aggregates were present in 62.5% of the FMCs, none were classified as score 3+ [15]. Later, Mills and colleagues (2015) categorized FMC inflammation into absent or very mild, predominantly lymphoplasmacytic and neutrophilic or pleocellular, but found no significant differences in the TSS of cats considering these categories. Nevertheless, felines with minimal inflammation had a slightly longer survival compared to those with lymphoplasmacytic infiltrate and, notably, they survived longer than those with neutrophilic or pleocellular infiltration [37]. Subsequent works have classified FMC peritumoral inflammation (i.e., lymphocytes, plasma cells, and macrophages) on a scale of 0 (absent) to 5 (severe), with score 3 representing moderate inflammation. Moderate inflammation was considered when the immune cells were observed in at least half of the tumor circumference, while marked (score 4) inflammation comprised the presence of multifocal inflammatory infiltrates along the entire tumor periphery, and severe inflammation (score 5) encompassed the presence of nodular lymphohistiocytic aggregates resembling lymphoid follicles. Moderate to severe peritumoral inflammation was observed in 47.3% to 57.2% of the FMC cases [28,107,108] and was significantly associated with poorer DFI, OS and TSS in univariate analysis [28,107], remaining a significant prognostic predictor for TSS in the multivariate analysis [107]. Although these later studies have addressed the inflammation in FMC, it was not the primary focus of their research, and a detailed characterization of this morphological feature was not provided. Additionally, these studies focused solely on malignant tumors, specifically carcinomas, leaving the inflammation associated with other mammary lesions, such as hyperplasias and benign neoplasms, unaddressed. Recently, our group carried out an overview of the inflammatory reaction in a large case series of feline mammary lesions—including non-neoplastic, benign and malignant lesions—and found that inflammation was more pronounced in malignant tumors. Moreover, the presence of inflammatory cells was associated with several clinicopathological features often linked to poor prognosis, thus highlighting the potential role of the inflammatory microenvironment in tumor initiation and progression [109].

By analyzing the methodology of the mentioned studies on FMC, it becomes clear that the methods for evaluating the inflammation are widely heterogeneous and ill-defined in the earlier studies, though more recent works have made an effort towards improving the definition of such methods. More recently, a couple of studies in FMC resorted to immunohistochemical techniques in order to better evaluate the immune microenvironment of these tumors [34,110], which will be addressed in the next sections. Notwithstanding, considering current literature in HBC and CMT, substantial investigation is still required regarding the evaluation of inflammation in FMTs.

### 6.1. Tumor-Infiltrating Lymphocytes

Lymphocytes play a pivotal role in adaptive immunity, and different lymphocyte subsets can take on paradoxical roles. For many years, lymphocytic immune responses have been characterized based on the T-helper cell (CD4+) subsets, which are classically differentiated into two distinct phenotypes, namely T-helper 1 (Th1) and T-helper 2 (Th2). However, like in many aspects of biology, recent findings indicate that the reality is more nuanced, as novel T-helper cell subsets, T-helper plasticity and beneficial mixed-type immune responses are uncovered [113]. In the context of cancer, Th1-predominant immune responses are typically linked to anti-tumoral activity, whereas Th2-prominent immune responses appear to favor tumor progression. Th1-type responses are associated with the secretion of interferon-gamma (IFN-ɣ), TGF-β, TNF-α, and interleukin (IL) 2, which upregulate cell cytotoxicity, thus supporting the activities of natural killer cells (NK), macrophages and cytotoxic T lymphocytes [114,115,116]. On the other hand, Th2-type responses are associated with the secretion of IL-4, IL-5, IL-6, IL-10 and IL-13, which downregulate cell cytotoxicity [114].

Among the most notable tumor-related Th1 and Th2 cytokines are IL-2 and IL-4, respectively [116]. Patients with breast cancer have been found to have significantly lower levels of circulating IL-2 and increased levels of circulating IL-4 when compared to healthy controls [117]. In another study, authors suggested that the increased circulating IL-4 may be influenced by hormone receptor status and might be linked to poorer outcomes in breast cancer [118]. Conversely, an independent case–control study observed significantly lower levels of circulating IL-4 in breast cancer patients, while IL-2 did not significantly differ from those of the controls [119]. In the canine species, a study evaluating circulating cytokine profiles in healthy controls, mammary carcinoma in benign mixed tumor and mammary carcinoma, reported that IL-2 was higher in the latter group. When observing the cytokine profiles of dogs with and without metastasis, IL-2 was increased in the “mammary carcinoma” group, irrespective of the metastasis status, while only dogs without metastasis in the “mammary carcinoma in the benign mixed tumor” group displayed an increase in this cytokine [120].

Taking on a broader view, the expression pattern of circulating cytokines in HBC seems to be different according to tumor clinical staging. Curiously, research found opposed dynamics between the local TME and systemic immune status, given that increased TILs within the TME were correlated to low pro-inflammatory circulating cytokine activity. On the other hand, local high FoxP3 regulatory T cells (Tregs) were positively correlated with high pro-inflammatory circulating cytokine status [121]. Taken together, these findings suggest that circulating cytokines might indirectly inform on the dynamics of the local TME, offering a non-invasive means to understanding cancer progression and immune responses. As research continues to explore the potential of measuring systemic inflammatory mediators, as well as how they relate to the local TME [121,122], this approach could enhance the ability to monitor disease progression and guide treatment decisions by providing more information with minimal patient burden in the routine clinical setting.

Several authors have focused their research on the study of TIL subsets in the context of HBC (Table S1). Accordingly, T lymphocytes are the most numerous immune cell subset, followed by macrophages, while B lymphocytes appear to be among the least frequent [123,124]. Even though high numbers of CD3+ T cells were associated with higher histological grade [124], increased amounts of intratumoral and stromal CD3+ TILs have been associated with improved survival and DFI [125]. Similarly, increased CD20+ B cells were associated with higher histological grade, but upon survival analysis, a high total count of this cell subset was an independent predictor for improved DFI and TSS. When separately analyzing different compartments, distant stromal B cells (CD20+) were also positively associated with better DFI; both intratumoral and stromal populations were independently linked to enhanced TSS [126]. Conversely, other authors found no relationship between CD20+ B cells and TSS. On the other hand, the presence of B cells with plasmacytic differentiation (CD138+) was an indicator of poorer DFI in invasive breast cancer [127].

Extensive research in HBC has been dedicated to the study of different T lymphocyte subsets. Notably higher numbers of CD4+, CD8+ and Tregs have been detected in the peritumoral region, rather than the tumoral region itself (tumor stroma and tumor nests) [128]. Cytotoxic T cells (CD8+) are well-known for their anti-tumoral role. Some studies associated the presence of higher counts of CD8+ TILs with higher histological grade [124,129], while others observed opposite results, with decreased numbers of CD8+ TILs being associated with clinicopathological parameters of aggressiveness, such as higher histological grade and increased Ki-67 proliferative index [130]. Overall, there is a consensus on the prognostic significance of this cell subset. Several authors found an association between increased CD8+ TILs and improved clinical outcomes (DFI and TSS), having remained a significant prognosis predictor after being adjusted to other clinicopathological features [124,127,129,131,132]. On the other hand, upon evaluation of separate compartments, tumors with positive intratumoral cytotoxic T cells were related to a poorer DFI in human patients with low-grade tumors, while both high intratumoral and stromal CD8+ TILs were associated with improved TSS in patients with high-grade tumors [129]. Similarly, intratumoral and stromal CD8+ TILs were an independent favorable prognostic factor for TSS in ER-negative tumors [131]. As opposed to cytotoxic T cells, Tregs typically play a pro-tumoral role. In human invasive breast cancer, the presence of higher counts of FoxP3+ Tregs was related to positive nodal status, higher histological grade and higher Ki-67 proliferative index [128,130,133]. Furthermore, increased Tregs have been linked to poorer outcomes pertaining to survival [123,124] and DFI [130], arising as an independent prognostic factor for DFI [133]. In another study, high ratios of Tregs to CD4+ and CD8+ TILs were associated with poor prognostic markers, viz high histological grade and tumor recurrence. Additionally, patients with high peritumoral Tregs and high Treg/CD8+ ratio showed lower 5-year DFI rates than patients with lower counts, with Treg/CD8+ ratio emerging as an independent predictor of DFI [128].

In FMCs, Nascimento and co-workers (2022) conducted a comprehensive evaluation of different lymphocyte subtypes within the intratumoral and stromal region of 73 tumors. Among these, CD3+ T cells were the most prevalent, followed by B cells and cytotoxic CD8+ T cells. Most T cell subsets were primarily found in the stroma, with the exception of CD8+ T lymphocytes, which were predominantly observed in the intratumoral region. Increased total and stromal CD3+ and CD4+ T cells were positively associated with higher tumor malignancy grade. Moreover, a higher density of intratumoral CD4+ T cells was significantly correlated with positive nodal status. Interestingly, while elevated intratumoral CD3+ T cells and stromal NK cells were positively linked to distant metastases, higher levels of total CD3+ and stromal CD8+ T cells showed a negative association with this feature. Furthermore, total CD8+ T cells were also associated with tumor necrosis, which is consistent with their role as a cytotoxic cell subset. Kaplan–Meier curves revealed that increased stromal CD8+ T cells were associated with significantly longer DFI and OS. In the univariate Cox regression, there was a significant relationship between stromal CD8+ T cells and OS. None of the other analyzed immune cell subtypes demonstrated a significant correlation with DFI or OS [34]. A couple of years prior, Urbano and co-workers (2020) reported the presence of interstitial FoxP3+ T cells in 74% of the evaluated FMCs, whereas no expression was observed in the normal control tissue [112]. In the same year, Dagher and colleagues (2020) provided a detailed assessment of Tregs in the intratumoral, stromal and peritumoral regions of 180 FMCs, particularly focusing on their relationship with clinicopathological features and prognostic value. The study found that increased numbers of Tregs in each region were positively associated with lymphohistiocytic tumor-associated inflammation and with each other. Intratumoral and stromal Tregs were more prevalent in tumors displaying squamous differentiation, with stromal Tregs also showing a positive correlation with LVI. Notably, a higher density of peritumoral Tregs was significantly associated with higher histological grade, presence of LVI, larger tumor size, positive nodal status and higher clinical stage. Moreover, the abundance of Tregs in any of the analyzed regions was linked to shorter DFI, OS and TSS in multivariate analysis [110].

### 6.2. Tumor-Infiltrating Macrophages

Macrophages are essential elements of the immune system, actively participating in both physiological and pathological processes. Specifically, macrophages contribute to mammary gland development at multiple stages and are key components of the breast TME [134]. Similarly to the points discussed in the previous section, TAMs can adopt both tumor-suppressing and tumor-promoting functions depending on the TME cytokine profile. Accordingly, a binary model of macrophage polarization is often referred to as representing the extremes of the TAM spectrum, namely classically activated (M1) macrophages, which are involved in Th1 responses and exhibit a pro-inflammatory anti-tumoral role, and alternatively activated (M2) macrophages, which are implicated in Th2 responses and display an anti-inflammatory pro-tumoral role. M1 macrophages function as antigen-presenting cells, priming T cells for adaptive anti-tumor responses. In addition, they produce nitric oxide (NO) and ROS, which are directly involved in the destruction of pathogens and tumor cells. M1 macrophages also secrete pro-inflammatory cytokines that help recruit and activate other immune cells, including cytotoxic T cells and NK cells. Conversely, M2 macrophages lose their antigen-presenting functions, instead contributing to an immunosuppressive TME. They produce anti-inflammatory cytokines that inhibit immune responses and participate in tissue remodeling and repair. Moreover, M2 macrophages support tumor progression by promoting angiogenesis through the secretion of VEGFs [134,135,136].

Tumor-associated macrophages have been extensively studied in HBC, revealing significant associations with clinicopathological features and patient outcomes (Table S2). Higher density of TAMs (CD68+) has been observed in cases with higher clinical stage, larger tumor size [137], higher histological grade, and higher Ki-67 proliferative index [138,139]. In addition, M2 macrophages (CD163+) were also significantly associated with high Ki-67 proliferative index and high histological grade [140]. Upon differential assessment of TAMs within the tumor stroma and tumor nests, a higher density of stromal CD68+, CD11c+ and CD163+ macrophages was positively associated with larger tumors [139,141], and denser stromal CD163+ macrophage infiltration was also associated with higher Ki-67 proliferative index and higher histological grade. On the other hand, intratumoral TAMs (CD68+ and CD163+) were not associated with clinicopathological features [141]. Within invasive breast cancer, the more aggressive invasive micropapillary carcinoma subtype exhibited dense infiltration of CD163+ macrophages. Furthermore, TAMs were predominantly seen along the invasive margin of these tumors, with higher numbers of CD68+ and CD163+ macrophages being associated with larger tumors and LVI, respectively [142]. Regarding the impact on survival, breast cancer patients with increased amounts of CD68+ TAMs displayed significantly poorer OS [137] and, particularly, intratumoral and stromal TAMs were significant predictors of poorer DFI [138] and TSS [141], respectively. M2 macrophages (CD163+) have also been reported as an independent predictor of DFI in breast cancer [140], specifically those in the intratumoral compartment [139]. In addition, increased infiltration of CD163+ TAMs was significantly associated with poorer OS and DFI in cases of invasive micropapillary carcinoma, and retained its significance pertaining to the DFI after multivariate analysis [142]. On the other hand, increased numbers of stromal M1 macrophages (CD11c+) were associated with favorable DFI and OS in cases of breast cancer, remaining an independent prognostic factor for DFI after multivariate analysis, while the same cell subset in the intratumoral compartment did not influence the clinical outcome [139]. Despite the overall acknowledgment of the importance of TAMs during carcinogenesis and progression, results are not always consensual among authors. While one study observed a significant relationship between abundant intratumoral CD68+ and CD163+ TAMs, and worse clinical outcomes (DFI and OS) [139], another study found no association between the same intratumoral cell subsets and DFI, OS and TSS [141]. The inverse situation was reported for increased stromal TAMs, which were linked to a poorer survival by Medrek and colleagues (2012), whereas Jeong and co-workers (2019) found no significant relationship when assessing the same features.

In FMCs, one study assessed the presence of CD68+ and CD163+ macrophages in the intratumoral and stromal compartments. While CD68+ macrophages were mostly found in the intratumoral region, the CD163+ macrophages prevailed within the tumor stroma. Total CD163+ macrophages were positively associated with high tumor malignancy grade [34].

### 6.3. Other Immune Cells

Myeloid-derived suppressor cells (MDSCs) are precursors of myeloid cells, both granulocytic and monocytic, that can be recruited into the TME. MDSCs contribute to TME immunosuppression by promoting Treg recruitment and activation, M2 macrophage polarization, while suppressing NK cells, CD4+ and CD8+ T cells. Moreover, MDSCs are associated with ROS production and the establishment of the pre-metastatic niche (PMN), and they have been implicated in therapeutic resistance, further emphasizing their tumor-promoting role in the TME. Multiple HBC studies link this cell population to unfavorable prognosis [12,143,144], warranting its research in FMCs.

Tumor-associated neutrophils (TANs), similar to macrophages, can polarize into tumor-suppressive N1 or tumor-promoting N2 phenotypes, with the latter usually prevailing as the tumor grows. TANs can contribute to tumor progression by producing angiogenic factors and matrix metalloproteinases, driving the continuous remodeling of the TME. In addition, TANs can also regulate the immune TME by suppressing T lymphocytes and NK cells, while recruiting Tregs, and they also release extracellular traps, which have been implicated in angiogenesis, tumor cell proliferation, adhesion, migration and metastasis [145,146]. In HBC, the peripheral neutrophil-to-lymphocyte ratio (NLR) has been proposed as a prognostic marker, with increased values associated with poor clinical outcomes, and potential relevance for therapeutic decision-making [145,147]. Similarly, in FMCs, high NLR has been associated with poor prognosis [148,149]; however, the role of neutrophils within the local FMC microenvironment remains unexplored.

Mast cells have a controversial role in cancer, including HBC. They can indirectly act as immunosuppressors in the TME via interactions with MDSCs and Tregs. Moreover, they have been implicated in the promotion of angiogenesis and lymphangiogenesis, tumor growth, invasion and metastasis [143,150]. On the other hand, other studies linked the presence of mast cells to a favorable prognosis, associating them with lower tumor grade, lower proliferation rates and hormone-receptive tumors. Furthermore, mast cells can exhibit cytotoxic activity and enhance tumor-inhibiting immune responses by recruiting NK cells, dendritic cells and T lymphocytes [150]. Only one study has evaluated mast cells in FMCs, using a small case–control series. This study reported increased numbers of intratumoral and peritumoral mast cells compared with healthy control tissues, with a predominance in the intratumoral compartment, regardless of the marker used (toluidine blue, tryptase or chymase). Furthermore, intratumoral mast cells were positively correlated with degranulated mast cells, suggesting an active role for this population in the FMC microenvironment rather than a passive bystander function [151].

Dendritic cells (DCs) are key antigen-presenting cells that phagocytose tumor antigens and subsequently deliver them to the draining lymph nodes, thereby regulating T cell differentiation and activating tumor-specific cytotoxicity [144,152]. The TME can compromise the anti-tumor functions of DCs, reducing their antigen-presenting activity and driving dysfunctional DCs to promote Treg proliferation, thereby contributing to poor prognosis and reduced responsiveness to immunotherapy [143,144,152]. Studies on DCs in HBC remain limited [144], and to the authors’ knowledge, no research has yet explored this cell population within the FMC microenvironment, highlighting the need for further investigation.

### 6.4. Immune Phenotypes

In recent years, distinct cancer immune phenotypes/profiles have been described based on the spatial patterns of the immune cells, such as immune-desert, immune-excluded and immune-inflamed [153]. The immune-desert or “cold” phenotype features a non-inflamed microenvironment, with scant lymphocytes in both the tumor parenchyma and stroma (tumor stroma and tumor periphery). This lack of lymphocytes suggests an absence of pre-existing anti-tumor immunity. The immune-excluded phenotype is characterized by an abundance of immune cells that are confined to the stroma adjacent to the tumor nests, without penetrating the tumor parenchyma itself. Thus, immune-excluded tumors are considered non-inflamed, given that the immune cells are not near the tumor cells. The immune-inflamed or “hot” phenotype is marked by the presence of immune cells in direct contact with the neoplastic cells. Both immune-excluded and immune-inflamed phenotypes indicate the presence of a pre-existing antitumor immunity [153,154].

The establishment of immune-desert and immune-excluded phenotypes arises from mechanisms that impair the efficacy of the anti-tumor responses, causing dysfunctions in tumor-cell antigen recognition and presentation, and hindrances in the function, migration and infiltration of T cells within the TME [154,155]. As such, this results in limited immunotherapeutic responses in these tumors, as opposed to the more responsive immune-inflamed phenotypes [155]. As personalized therapeutic approaches gain relevance, recent efforts have focused on converting immune-excluded and immune-desert TMEs into immune-inflamed phenotypes, seeking to address the underlying mechanisms of immune evasion and exclusion, and improving the treatment response of previously resistant tumors [154,155].

In line with the concept of cancer immune phenotype, the importance of the spatial distribution of immune cells has been recently highlighted in a study on FMC, after the authors observed that elevated total CD3+ lymphocytes were inversely related to metastasis, while increased intratumoral CD3+ TILs were linked to the presence of tumor metastasis [34]. Interestingly, prior research in CMT revealed the presence of higher numbers of intratumoral CD3+ lymphocytes in benign CMT, as opposed to malignant tumors, in which a greater count of CD3+ was confined to the peritumoral compartment [156]. These spatial patterns might suggest that immune evasion mechanisms similar to those reported in the context of human oncology may take place during domestic animal mammary tumorigenesis.

### 6.5. Immune Checkpoints

Immune checkpoints are pivotal regulators of the immune system, acting as inhibitory signals that limit overactive immune responses and preserve self-tolerance. These are particularly important in the context of HBC, as therapeutic approaches involving immune checkpoint inhibitors have the potential to improve anti-tumor immune responses [157]. Recent works have begun to investigate several immune checkpoint molecules in FMCs, namely PD-1 and PD-L1, CTLA-4, lymphocyte activation gene 3 (LAG-3), mucin-domain-containing molecule-3 (TIM-3) and V-domain immunoglobulin suppressor of T cell activation (VISTA) [112,158,159,160].

The expression of PD-L1 has been detected in feline macrophage and FMC cell lines [161], and PD-1, PD-L1 and PD-L2 have been identified in TILs and tumor cells of spontaneous FMCs [158,162]. PD-1+ and PD-L1+ TILs were reported in 41.7% and 66.7% of the HER2-positive FMCs, respectively, whereas the same biomarkers were detected in 33.3% and 20% of the triple-negative normal-like tumors, respectively [158]. In a case series of rare FMT histological subtypes, PD-1+ intratumoral and stromal TILs were found in 85% and 94% of tumors, respectively. Similarly, PD-L1+ TILs were observed in 96% of intratumoral and 100% of stromal tumor areas, while PD-L2+ TILs were detected in all cases across both compartments [162]. PD1+ and PD-L1+ cancer cells were detected in 100% of FMCs, irrespective of the tumor molecular subtype [158], whereas in rare FMT histotypes, results differed, with PD-L1+ cancer cells identified in 46% of cases [162]. Moreover, increased serum levels of PD-1 and PD-L1 were observed in cats with HER2-positive and triple-negative normal-like carcinomas compared with healthy controls [158].

VISTA expression has also been identified in both tumor cells and TILs of FMCs. Positivity to VISTA was reported in the TILs and cancer cells of 82.6% and 100% of cases, respectively. The HER2-positive subtype had greater VISTA+ TILs than triple-negative tumors, and higher VISTA+ tumor cells were associated with metastasis in the former subtype. At the systemic level, serum VISTA concentrations were higher in cats with luminal A, HER2-positive and triple-negative mammary carcinomas compared with healthy controls, with increased levels further correlating with metastasis in the luminal A subtype [159].

CTLA-4+ TILs were observed in 69.4% of FMCs, while only 4.1% of tumor cells were positive for the marker. Increased serum CTLA-4 levels were present in FMCs compared with healthy controls [112].

TIM-3 expression was observed in stromal and intratumoral TILs in 93.3% and 64.4% of FMCs, and was also observed in tumor cells. Interestingly, the prevalence of intratumoral TIM-3+ TILs progressively decreased from luminal A to luminal B, HER2-positive and, finally, triple-negative carcinomas, while stromal TIM-3+ TILs expression remained predominantly consistent across subtypes, aside from a slight reduction in HER2-positive tumors. In addition, while the percentage of TIM-3+ intratumoral TILs was negatively correlated with tumor size and clinical stage, the percentage of TIM-3+ stromal TILs was positively correlated with histological grade and solid histological subtype. Moreover, cats with triple-negative carcinomas exhibiting increased TIM-3+ stromal TILs had significantly better clinical outcomes than those with lower TIM-3+ stromal TILs. Increased percentages of TIM-3+ cancer cells were also associated with nodal metastasis, luminal B and triple-negative basal-like subtypes. Overall, serum TIM-3 concentrations were significantly reduced in FMCs compared with healthy controls. Lower levels were associated with shorter DFI, and serum TIM-3 showed a positive correlation with TIM-3 expression in the TME [160].

Notably, positive correlations have been reported between several immune checkpoint molecules in FMC cases, namely serum VISTA, PD-1, PD-L1, CTLA-4, LAG-3, and cytokines IL-6 and TNF-α [112,158,159,160].

Most of the aforementioned studies have not conducted survival analyses, leaving the prognostic value of immune checkpoint molecules in FMC largely unclear. Nevertheless, current evidence offers insights into the local and systemic immune status of FMC and the interplay between circulating and tumor-infiltrating components. These findings not only support the relevance of FMC as a comparative model for HBC but also establish a foundation for exploring novel immunotherapeutic strategies [112,157,159,160]. From a therapeutic standpoint, integrating immune checkpoint inhibitors with other complementary strategies—such as anti-angiogenic and anti-fibrotic/CAF approaches—may help remodel the TME and improve treatment efficacy. In particular, immune checkpoint inhibitors hold promise for converting “cold” tumor immune phenotypes with limited TIL infiltration into “hot” tumors, thereby enhancing responsiveness to immunotherapy and opening new therapeutic avenues for both veterinary and human oncology.

## 7. Extracellular Vesicles

Extracellular vesicles (EVs) are membrane-bound particles secreted by cells under both physiological and pathological conditions, and they can be detected in all body fluids. These are typically representative of their cell of origin, serving as vehicles for intercellular communication by transporting nucleic acids, proteins, lipids, metabolites and cytokines. Over the past few years, the role of EVs in tumorigenesis and therapeutic resistance has garnered substantial attention from the scientific community [163,164,165]. Studies have shown that both cancer- and stromal cell-derived EVs can transport molecules involved in tumor cell proliferation and survival, angiogenesis, cell migration, invasion and metastasis [164]. Notably, EVs have been implicated in the development of metastatic lesions and as mediators of the communication between the primary tumor and the PMN [164]. As the primary tumor grows, the hypoxic and inflammatory milieu gives rise to the secretion of tumor-derived factors and EVs into the circulation to establish the PMN in distant organs, by promoting a local immunosuppressive microenvironment [166].

In FMC, EVs were first isolated and characterized in 2018 [167]. A subsequent review on EVs as biomarkers of both HBC and FMC drew parallels between the two species, with particular emphasis on triple-negative breast cancer, underscoring the potential of EV research in this comparative model [165]. Later, one study on EVs in FMC detected differences in the expression of microRNAs-20a and -15b between the tumor and its margins. Moreover, plasma EVs from diseased cats had significantly lower levels of these microRNAs and different proteomic profiles compared to healthy controls, allowing the distinction between the two groups and supporting their potential as non-invasive diagnostic biomarkers of FMC [168]. Another study observed that the main target tissues of FMC-derived EVs were the primary tumor and the liver, with tumor cells and hepatic stellate cells (HSCs) as the main cellular recipients. Moreover, sphingosine kinase 1 (SK1) carried by FMC-derived EVs was shown to be essential for establishing the hepatic PMN by activating HSCs via signal transducer and activator of transcription 3 (STAT3) [169]. The same group had previously reported the presence of SK1 in FMCs and its association with aggressive tumor features, such as higher histological grade, LVI and nodal metastases [170]. Taken together, these findings suggest that SK1 may be a potential molecular target for FMC therapy [169,170]. Although the field of EVs on FMC remains largely unexplored and at an embryonic stage, research reveals considerable potential, warranting further exploration.

## 8. Epithelial–Mesenchymal Transition

The epithelial–mesenchymal transition (EMT) is a typically reversible process during which epithelial cells acquire mesenchymal traits. Accordingly, epithelial cells go through cytoskeleton changes, lose their characteristic apical-basal polarity and intercellular adhesion, while gaining increased motility and invasiveness [55,171,172]. This process allows tumor cells to invade and metastasize to distant locations [55,172].

In one study assessing the mRNA expression of recognized EMT-related transcription factors, snail family transcriptor repressor 1/2 (SNAIL), TWIST 1/2, zinc-finger-enhancer binding protein 1/2 (ZEB) were all downregulated in FMCs compared to healthy controls [173]. These results align with prior observations of diminished TWIST 1 expression in FMCs [174]. The same study also evaluated EMT-associated proteins, showing negative associations between membranous and cytoplasmic expression of E-cadherin and between cytokeratin 5/6 and vimentin expression. High immunoreactivity for vimentin was detected in FMCs and triple-negative HBC, underscoring the aggressive behavior shared by these tumors [173]. This observation is consistent with earlier reports in which vimentin staining was identified in the epithelial cells of up to 76% of FMCs [175,176]. One study on an FMT with anaplastic and spindle cells also revealed co-expression of epithelial and vimentin markers in both tubular and solid anaplastic tumor regions [177].

Over the years, several studies have focused on adhesion molecules in FMTs. P-cadherin was aberrantly expressed in the luminal epithelial cells of up to 80.0% of malignant tumors, whereas in normal mammary glands and benign lesions, its expression was largely confined to myoepithelial cells. Additionally, increased P-cadherin expression was observed in the most invasive tumor regions, suggesting a role in promoting tumor cell invasiveness. Notably, P-cadherin overexpression was linked to aggressive histological features—including higher histological grade, necrosis, infiltrative tumor growth and LVI—further supporting that the molecule may serve as a marker of poor prognosis in FMCs [178,179]. By contrast, E-cadherin was significantly reduced (≤75% positive cells) in 46.7% of malignant tumors compared to normal mammary tissues and benign tumors [179]. One study reported a particularly reduced and weaker E-cadherin staining in solid regions and in large, poorly differentiated tumor cells [180], while another reported an absence of immunoreaction in anaplastic cells [177]. Upon evaluation of the combined expression pattern of P-cadherin and E-cadherin, none of the FMTs with LVI and/or nodal metastases had reduced immunoreactivity of P-cadherin, while some maintained E-cadherin expression [179]. The cadherin-catenin complex has also been assessed in FMTs. One study comparing carcinomas with normal mammary tissues and benign tumors reported that 70% of malignant tumors had underexpression of at least one molecule—E-cadherin, α-, β- and p120-catenin. Moreover, 60% of tumors with diminished immunoreaction of at least one catenin also displayed reduced E-cadherin [181]. Similar findings were described in earlier studies, including one that linked decreased E-cadherin and β-catenin expression with nodal metastases [175,182]. Additionally, a pattern of weaker immunoreaction for E-cadherin and β-catenin was observed in the most infiltrative portions of FMCs [182]. However, the prognostic value of E-cadherin and β-catenin appeared to be limited, as neither molecule was significantly associated with survival outcome [182]. CD44 has also been investigated by a few studies on FMTs. High CD44 immunoreactivity was reported in intratumoral areas compared with extratumoral regions [60], as well as in anaplastic and spindle tumor cells [177]. In contrast, other authors have described low CD44 expression in FMCs [173].

A recent study explored the detection of EMT-related changes in cytological samples by using a fluorescence immunocytochemistry technique. Among the four included FMCs, all showed reduced E-cadherin expression alongside vimentin positivity. These findings were confirmed by immunohistochemistry, suggesting that fluorescence immunocytochemistry may be a potential non-invasive approach for evaluating EMT in FMCs [183].

Taken together, studies on FMTs reveal consistent changes in both EMT-related transcription factors and adhesion molecules. In particular, reduction in molecules associated with the cadherin-catenin complex, in conjunction with vimentin overexpression, reflects a shift toward a mesenchymal phenotype. These findings underscore the contribution of EMT to the highly invasive and metastatic behavior of FMCs.

## 9. Conclusions

Studies from HBC provide invaluable context for understanding the feline TME; however, it is important to acknowledge that this field displays complexities and controversies. Several components of the TME, particularly immune cell populations, can adopt dual roles, shifting between tumor-promoting and tumor-inhibiting functions in response to microenvironmental cues. Recognizing this plasticity provides a more balanced perspective on the TME and further underscores the need for dedicated studies in FMC to clarify how such dynamics manifest in cats. Although many studies addressed the TME, particularly the inflammatory TME, in HBC and, to a lesser extent, in CMT, studies specifically focusing on FMTs remain very limited. Addressing these knowledge gaps will enhance our understanding of the pathogenesis and progression of these tumors and may contribute towards the development of more effective therapeutic strategies focusing on specific targets of the TME.

Current prognostic assessment of FMC relies heavily on traditional clinical and histopathological features, which provide only a partial picture of the tumor. While these features rely almost exclusively on the neoplastic cells, we now know that TME comprises fibroblasts, immune cells, endothelial cells, adipocytes and ECM, all engaging in an intricate dialogue that continuously modulates the tumor behavior. Over the years, cancer research has been shifting its focus from a tumor cell-centric model to a broader TME-centric framework, in search of more effective and individualized treatment strategies that are able to tip the scale in favor of a tumor suppressive microenvironment. The TME remains largely unexplored in FMCs, with few studies addressing some of its constituents as their main focus. The scarcity of studies dedicated to the TME of FMCs creates a significant gap in knowledge, restricting the ability to draw robust conclusions and highlighting the TME as a critical avenue for future research. Moreover, current FMC treatment is usually surgery alone, as the beneficial effects provided by chemotherapy in this species are still limited. A deeper understanding and characterization of the FMC microenvironment may not only help us refine prognostic assessments but also provide opportunities for novel and more efficient treatment strategies, as additional potential therapeutic targets come to light. Additionally, given the clinical and pathological similarities between FMC and HBC, along with the fact that cats are immunocompetent animals that share the same environment as humans and have naturally occurring mammary tumors, the study of FMC may provide new insights into tumor progression, tumor-associated immune responses and therapeutic responses in ways traditional rodent models may not fully replicate.

Several studies in FMCs have begun exploring circulating biomarkers in parallel to the local TME, suggesting that these peripheral markers could have value as non-invasive diagnostic and prognostic tools, as well as possible guides for therapeutic strategies. Alongside this line of research, future studies on FMTs should prioritize the development of standardized and reproducible methods for evaluating TME components. In this sense, research on HBC, where such systems are more advanced, can provide valuable guidance for its veterinary counterparts. As oncology moves toward precision medicine, conventional pathology practices must evolve and adapt to this new era by incorporating novel diagnostic tools, such as tissue-based multiplexing, spatial transcriptomics and digital image analysis. These approaches enable a detailed assessment of co-expression profiles, spatial arrangements and respective interactions, offering a more accurate prediction of therapeutic responses [184,185,186]. With these methods only beginning to gain traction in human oncology, their use in veterinary oncology may be distant; however, awareness and gradual integration of these innovations will be essential to align future FMC research with broader advances in comparative cancer biology.

## Figures and Tables

**Table 1:** Summary of studies on feline mammary carcinomas, including survival analyses of markers related to tumor necrosis, angiogenesis, fibrosis and adipose tissue.

Marker (Technique)	Categories/Thresholds	Significance	Outcome	Statistical Model	Reference
**Tumor necrosis**					
Necrosis (HE)	Absence, some necrosis, ≈30%, 60%, ≥90%	Yes	OS	MV	[39]
	25% cutoff	Yes	1-year OS	UV	[27]
	Absent, isolated cells, extensive	No	TSS/DFI	UV	[38]
	25% cutoff	No	TSS	UV	[37]
	Central necrosis of any type: present, absent	No	OS/TSS	UV	[28]
	Ischemic central necrosis: present, absent	No	OS/TSS/DFI	UV	[35]
	Comedo necrosis: present, absent Random necrosis: present, absent 25% cutoff	No	OS/TSS/DFI	UV	
Necrosis (HE stereology)	0.130 cutoff	No	OS/TSS/DFI	UV	
HIF-1α (IHC)	Overexpression: nuclear immunoreactivity in any tumor cells	Yes	1-year OS	UV	[27]
**Tumor angiogenesis**					
VEGF (IHC)	72.1% cutoff	Yes	OS	UV	[44]
	Score 0 (absence of staining), score 1 (weak immunoreactivity in <50% of tumor cells), score 2 (weak immunoreactivity in ≥50% or strong immunoreactivity in <50% of tumor cells), score 3 (strong immunoreactivity in ≥50% of tumor cells) Overexpression: scores 2 and 3	No	TSS	UV	[45]
		No	1-year OS	UV	[27]
MVD (IHC)	29.5 cutoff	No	OS	UV	[44]
**Tumor fibrosis**					
Collagen fibers (SHG) *	Collagen density (mean cutoff)	Yes	OS/DFI	UV	[46]
	Tumor-stroma boundary: N (not enough collagen to score), score 0 (distinct division), score 1 (discrete) Tumor-stroma boundary (mean cutoff)	Yes	OS/DFI	UV	
		Yes	OS/DFI	MV	
	Collagen fiber number (mean cutoff)	No	OS/DFI	UV	
		Yes	OS/DFI	MV	
	Collagen fiber length (mean cutoff)	Yes	OS/DFI	UV	
	Collagen fiber width (mean cutoff)	Yes	OS/DFI	UV	
		Yes	DFI	MV	
	Collagen fiber straightness (mean cutoff)	Yes	OS/DFI	UV	
		Yes	DFI	MV	
Stromal response (HE)	None to mild, peritumoral, intratumoral	Yes	1-year OS	UV	[27]
α-SMA (IHC)	Immunoreactivity percentage: score 0 (absence of staining), score 1 (1–10% positive CAFs), score 2 (11–50% positive CAFs), score 3 (51–80% positive CAFs), score 4 (81–100% positive CAFs) Immunoreactivity intensity: score 0 (no staining), score 1 (weak), score 2 (moderate), score 3 (intense/strong) Final score = percentage score + intensity score Low immunoreactivity: final score < 6; high immunoreactivity: final score ≥ 6	Yes	TSS/DFI	UV	[47]
**Adipose tissue**					
Leptin (ELISA)	4.17 pg/mL cutoff	Yes	DFI	UV	[32]

HE—Hematoxylin and eosin. IHC—Immunohistochemistry. SHG—Second-harmonic generation. ELISA—Enzyme-linked immunosorbent assay. HIF-1α—Hypoxia-inducible factor-1 alpha. VEGF—Vascular endothelial growth factor. MVD—Microvessel density. α-SMA—Alpha smooth muscle actin. CAFs—Cancer-associated fibroblasts. OS—Overall survival. TSS—Tumor-specific survival. UV—Univariate. MV—Multivariate. * Both continuous and categorical variables were tested for each parameter; herein, only the results for the categorical variables are shown.

**Table 2:** Summary of feline malignant mammary tumor studies, including evaluation of the tumor-associated inflammatory reaction.

*n*	Categories		% of Cases	Survival Times (Median)	Significance	Survival Variable	Statistical Model	Reference
170	Lymphocytes and plasma cells		-	-	No	-	-	[106]
202	Quantity of acute and chronic cellular inflammation		-	-	Yes	OS	UV	[39]
					No	OS	MV	
24	Absent to negligible		37.5%	-	-	-	-	[15]
	Few moderate peritumoral lymphoid aggregates		41.7%	-	-	-	-	
	Moderate peritumoral lymphoid aggregates		20.8%	-	-	-	-	
108	Absent or mild		31.5%	18 months	No	TSS	UV	[37]
	Lymphoplasmacytic		54.6%	15 months				
	Neutrophilic or pleocellular		13.9%	9 months				
342	Mononuclear	Absent to mild	48.5%	13.2 months	Yes	OS/TSS	UV	[28]
		Moderate to severe	51.4%	8.5 months				
180	Mononuclear	Absent to mild	42.8%	-	-	-	-	[108]
		Moderate to severe	57.2%					
395	Absent to mild (mononuclear)		52.7%	-	Yes	DFI/OS/TSS	UV	[107]
	Moderate to severe (mononuclear)		47.3%			TSS	MV	
180	Intratumoral Tregs (≥2 vs. <2/mm^2^)		82.2%	-	Yes	DFI/OS/TSS	MV	[110]
	Stromal Tregs (≥6 vs. <6/mm^2^)		91.7%			DFI/OS/TSS	MV	
	Peritumoral Tregs (≥575 vs. <575/mm^2^)		97.2%			DFI/OS/TSS	UV/MV	
10	Peripheral lymphocytes		80.0%	-	-	-	-	[111]
49	Positive score interstitial FoxP3+ lymphocytes		71.4%	-	-	-	-	[112]
	None seen		14.3%	-	-	-	-	
	<1% of FoxP3+ interstitial lymphocytes		8.2%	-	-	-	-	
	1–5% of FoxP3+ interstitial lymphocytes		42.9%	-	-	-	-	
	6–30% of FoxP3+ interstitial lymphocytes		26.5%	-	-	-	-	
	>30% of FoxP3+ interstitial lymphocytes		8.2%	-	-	-	-	
40	Lymphocytic infiltration (yes vs. no)		67.5%	-	-	-	-	[31]
56	Lymphocytic infiltration (yes vs. no)		66.1%	-	-	-	-	[32]
73	Lymphocytic infiltration (yes vs. no)		78.1%	-	-	-	-	[34]
	Intratumoral, stromal and total cells: CD3+, CD4+ and CD8+ * T cells, Tregs, B cells, NK cells, CD68+ and CD163+ macrophages		-	-	No	DFI/OS	UV	
	Low stromal CD8+ T cells		-	15.5 months	Yes	DFI/OS	UV	
	High stromal CD8+ T cells		-	31.0 months				
150	Peritumoral inflammation distribution	Absent from focal	5.3%	-	-	-	-	[109]
		Multifocal to diffuse	94.7%	-	-	-	-	
	Intratumoral inflammation distribution	Absent from focal	28.7%	-	-	-	-	
		Multifocal to diffuse	71.3%	-	-	-	-	
	Perilesional inflammation intensity	Discrete	34.2%	-	-	-	-	
		Moderate to marked	65.8%	-	-	-	-	
	Intratumoral inflammation intensity	Discrete	58.2%	-	-	-	-	
		Moderate to marked	41.8%	-	-	-	-	
	Tertiary lymphoid structures (present vs. absent)		11.7%	-	-	-	-	

OS—Overall survival. TSS—Tumor-specific survival. DFI—Disease-free interval. UV—Univariate. MV—Multivariate. Tregs—Regulatory T cells. NK—Natural killer cells. * Stromal CD8+ T cells showe a statistically significant associaton with overall survival.

## Data Availability

No new data were created or analyzed in this study. Data sharing is not applicable to this article.

## Supplementary Materials

The following supporting information can be downloaded at: https://www.mdpi.com/article/10.3390/vetsci12100959/s1, Table S1: Summary of studies on tumor-infiltrating lymphocytes in human breast cancer; Table S2: Summary of studies on tumor-infiltrating macrophages in human breast cancer.
