# Supplementary material for: Inside the Tumor: Decoding the Feline Mammary Tumor Microenvironment and Its Prognostic Value—A Review
Source: Vet Sci. 2025 Oct 8;12(10):959. doi: 10.3390/vetsci12100959 (PMC12568044; doi:10.3390/vetsci12100959)
Supplement: Supplementary file 1 [file vetsci-12-00959-s001.zip › vetsci-3858921-supplementary.pdf]

Table S1. *Summary of studies on tumor-infiltrating lymphocytes in human breast cancer.*

n	Categories	Location	Cell population	Significance	Endpoint	Statistical model	Reference
222	High *vs* low (median 15 positive cells cutoff)	Intratumoral (stroma and tumor nests)	FoxP3+	Yes	OS/DFI	UV	(Bates et al., 2006)
				Yes	DFI	MV	
				Yes	OS/DFI (ER-positive)	UV/MV	
				No	OS/DFI (ER-negative)	UV	
1953	High *vs* low (0 positive cells cutoff)	Intratumoral	CD8+	No	TSS	UV	(Baker et al., 2011)
				Yes	TSS (ER-negative)	UV	
				Yes	TSS (ER-positive)	UV	
				No	TSS (ER-positive)	MV	
	High *vs* low (2 positive cells cutoff)	Stromal		No	TSS	UV	
				Yes	TSS (ER-negative)	UV	
	High *vs* low (1 positive cells cutoff)	Total (stroma and tumor nests)		No	TSS	UV	
				Yes	TSS (ER-negative)	UV/MV	
1470	High *vs* low (1 positive cells cutoff)	Intratumoral	CD20+	No	TSS/DFI	UV	(Mahmoud et al., 2012)
				Yes	TSS/DFI	MV	
		Stromal (adjacent)		No	TSS/DFI	UV	
				No	DFI	MV	
				Yes	TSS	MV	
	High *vs* low (5 positive cells cutoff)	Stromal (distant)		Yes	DFI	UV	
				No	TSS	UV	
				Yes	TSS/DFI	MV	
		Total		Yes	TSS/DFI	UV/MV	
72	High *vs* low (60 positive cell count value cutoff)	Intratumoral (stroma and tumor nests)	CD8+	No	DFI	UV	(S. T. Kim et al., 2013)
	High *vs* low (17 positive cell count value cutoff)		FoxP3+	Yes	DFI	UV	
				No	DFI	MV	
338	Tertiles	Not specified	CD4+	No	TSS	UV	(Mohammed et al., 2013)
			CD8+	Yes	TSS	UV	
				Yes	DFI	UV	
			CD138+	Yes	TSS/DFI	UV	
			CD20+	No	TSS	UV	

	High *vs* low		TILs	Yes	DFI	UV	
				Yes	TSS	UV/MV	
127	High *vs* low<br>(25 positive cells cutoff)	Intratumoral and stromal	CD3+	Yes	OS/DFI	UV	(Rathore et al., 2013)
12439	Positive *vs* negative<br>(0 positive cells cutoff)	Intratumoral	CD8+	Yes	TSS (ER-positive)	UV	(Ali et al., 2014)
				Yes	TSS (ER-negative	UV/MV	
				No	TSS (ER-positive)	MV	
		Stromal		Yes	TSS (ER-negative)	UV/MV	
				No	TSS (ER-positive)	UV	
		Intratumoral	FoxP3+	Yes	TSS (ER-positive)	UV	
				No	TSS (ER-negative)	UV	
				No	TSS (ER-positive)	MV	
		Stromal		No	TSS (ER-positive, ER-negative)	UV/MV	
332	Positive *vs* negative<br>(0 positive cells cutoff)	Intratumoral	CD8+	Yes	TSS/DFI	UV/MV	(Z. Chen et al., 2014)
				Yes	TSS/DFI (ER-negative, HER2-negative, TNBC)	UV	
				No	TSS/DFI (ER-positive, HER2-positive)	UV	
	Intensity score pairs<br>(0, 1+, 2+, 3+)	Peritumoral		No	TSS/DFI	UV	
143	High vs low<br>(average cell count value cutoff)	Intratumoral (stroma and tumor nests) and peritumoral	CD4+	No	OS/DFI	UV	(S. Kim et al., 2014)
		Intratumoral (stroma and tumor nests) and peritumoral	CD8+	No	OS/DFI	UV	
		Intratumoral	FoxP3+	No	OS/DFI	UV	
		Peritumoral	FoxP3+	Yes	DFI	UV	
				No	OS	UV	
1165	High *vs* low<br>(10% score cutoff)	Not specified	CD3+	Yes	DMFS (TNBC)	UV	(Althobiti et al., 2018)
				Yes	TSS (TNBC)	UV/MV	
			CD20+	Yes	TSS (TNBC, HER2-positive)<br>DMFS (TNBC)	UV	
				No	TSS	UV/MV	
			CD8+	Yes	DMFS (TNBC)	UV	
				Yes	TSS (TNBC)	UV/MV	

			FoxP3+	Yes	TSS	UV	
				No	TSS	MV	
			CD68+	Yes	TSS<br>TSS (luminal B)	UV	
				No	TSS	MV	
	Average HE TILs	Stromal	HE TILs	No	TSS	UV	
			HE TILs	Yes	TSS (TNBC)	UV/MV	
	Hotspot HE TILs		HE TILs	No	TSS/DMFS	UV	
485	High *vs* low<br>(median cell count value cutoff)	Stromal	CD3+	No	OS/LR/DFI	UV	(Millar et al., 2020)
			CD20+	Yes	OS<br>OS (luminal)	UV/MV	
				Yes	OS (TNBC)	UV	
				No	LR/DFI	UV	
			CD8+	Yes	OS/LR/DFI<br>OS/DFI (luminal)	UV	
				Yes	LR/DFI<br>DFI (luminal)	MV	
			FoxP3+	Yes	OS<br>OS (TNBC)	UV	
				No	LR/DFI	UV	
			CD68+	No	OS/LR/DFI	UV	
			HE TILs	Yes	OS	UV	
				No	LR/DFI	UV	

UV – Univariate. MV – Multivariate. DMFS – Distant metastases-free survival. LR – Local recurrence. TNBC – Triple-negative breast cancer.

Table S2. *Summary of studies on tumor-infiltrating macrophages in human breast cancer.*

n	Categories	Location	Cell population	Significance	Endpoint	Statistical model	Reference
144	Absent/sparse *vs* dense	Intratumoral	CD68+	No	OS/TSS/DFI	UV	(Medrek et al., 2012)
144	Absent/sparse *vs* dense	Stromal	CD68+	Yes	OS/TSS	UV	(Medrek et al., 2012)
144	Absent/sparse *vs* dense	Stromal	CD68+	Yes	TSS	MV	(Medrek et al., 2012)
144	Absent/sparse *vs* dense	Stromal	CD68+	No	OS/DFI	MV	(Medrek et al., 2012)
144	Absent/sparse *vs* dense	Intratumoral	CD163+	No	OS/TSS/DFI	UV	(Medrek et al., 2012)
144	Absent/sparse *vs* dense	Stromal	CD163+	Yes	OS/TSS<br>OS (luminal A)	UV	(Medrek et al., 2012)
144	Absent/sparse *vs* dense	Stromal	CD163+	No	OS (TNBC basal-like)	UV	(Medrek et al., 2012)
144	Absent/sparse *vs* dense	Stromal	CD163+	No	OS/TSS/DFI	MV	(Medrek et al., 2012)
276	High *vs* low (median cutoff)	Intratumoral	CD68+	Yes	DFI<br>DFI (HR-positive)	UV/MV	(Gwak et al., 2015)
276	High *vs* low (median cutoff)	Intratumoral	CD68+	No	DFI (HR-negative)	UV	(Gwak et al., 2015)
276	High *vs* low (median cutoff)	Stromal	CD68+	No	DFI	UV	(Gwak et al., 2015)
276	High *vs* low (median cutoff)	Stromal	CD68+	Yes	DFI (HR-positive)	UV	(Gwak et al., 2015)
276	High *vs* low (median cutoff)	Total (intratumoral and stromal)	CD68+	No	DFI<br>DFI (HR-negative)	UV	(Gwak et al., 2015)
276	High *vs* low (median cutoff)	Total (intratumoral and stromal)	CD68+	Yes	DFI (HR-positive)	UV	(Gwak et al., 2015)
562	Not specified	Total	CD68+	No	OS/DFI	UV	(Sousa et al., 2015)
562	High *vs* low (167 positive cells cutoff)	Total	CD163+	No	OS/DFI	UV	(Sousa et al., 2015)
562	High *vs* low (167 positive cells cutoff)	Total	CD163+	Yes	DFI	MV	(Sousa et al., 2015)
100	High *vs* low (median 61 positive cells cutoff)	Intratumoral	CD68+	Yes	OS	UV	(J. Yang et al., 2015)
222	Positive *vs* negative (10% staining cutoff)	Stromal	CD68+	No	OS/DFI	UV	(Liu et al., 2017)
222	Positive *vs* negative (10% staining cutoff)	Stromal	CD163+	Yes	OS/DFI	UV	(Liu et al., 2017)
222	Positive *vs* negative (10% staining cutoff)	Stromal	CD163+	Yes	DFI	MV	(Liu et al., 2017)
367	High *vs* low (33 positive cells cutoff)	Intratumoral	CD68+	Yes	OS/DFI	UV	(Jeong et al., 2019)
367	High *vs* low (17.8 positive cells cutoff)	Stromal	CD68+	No	OS/DFI	UV	(Jeong et al., 2019)
367	High *vs* low (1.67 positive cells cutoff)	Intratumoral	CD163+	Yes	OS/DFI	UV	(Jeong et al., 2019)
367	High *vs* low (1.67 positive cells cutoff)	Intratumoral	CD163+	Yes	DFI	MV	(Jeong et al., 2019)
367	High *vs* low (21 positive cells cutoff)	Stromal	CD163+	No	OS	UV	(Jeong et al., 2019)
367	High *vs* low (20 positive cells cutoff)	Stromal	CD163+	No	DFI	UV	(Jeong et al., 2019)
367	High *vs* low (1 positive cells cutoff)	Intratumoral	CD11c+	No	OS	UV	(Jeong et al., 2019)

	High *vs* low (10 positive cells cutoff)			No	DFI	UV	
	High *vs* low (75 positive cells cutoff)	Stromal		Yes	OS/DFI	UV	
				Yes	DFI	MV	

UV – Univariate. MV – Multivariate. HR – Hormone receptor
